# The therapeutic potential of non-invasive brain stimulation for the treatment of Long-COVID-related cognitive fatigue

**DOI:** 10.3389/fimmu.2022.935614

**Published:** 2023-01-09

**Authors:** Stefanie Linnhoff, Lilli Koehler, Aiden Haghikia, Tino Zaehle

**Affiliations:** ^1^ Department of Neurology, Otto-von-Guericke-University Magdeburg, Magdeburg, Germany; ^2^ Center for Behavioral Brain Sciences (CBBS), Magdeburg, Germany

**Keywords:** Long-COVID, Post-COVID, cognitive fatigue, fatigability, NIBS, tDCS, tACS, tVNS

## Abstract

Following an acute COVID-19 infection, a large number of patients experience persisting symptoms for more than four weeks, a condition now classified as Long-COVID syndrome. Interestingly, the likelihood and severity of Long-COVID symptoms do not appear to be related to the severity of the acute COVID-19 infection. Fatigue is amongst the most common and debilitating symptoms of Long-COVID. Other symptomes include dyspnoea, chest pain, olfactory disturbances, and brain fog. Fatigue is also frequently reported in many other neurological diseases, affecting a broad range of everyday activities. However, despite its clinical significance, limited progress has been made in understanding its causes and developing effective treatment options. Non-invasive brain stimulation (NIBS) methods offer the unique opportunity to modulate fatigue-related maladaptive neuronal activity. Recent data show promising results of NIBS applications over frontoparietal regions to reduce fatigue symptoms. In this current paper, we review recent data on Long-COVID and Long-COVID-related fatigue (LCOF), with a special focus on cognitive fatigue. We further present widely used NIBS methods, such as transcranial direct current stimulation, transcranial alternating current stimulation, and transcutaneous vagus nerve stimulation and propose their use as possible therapeutic strategies to alleviate individual pathomechanisms of LCOF. Since NIBS methods are safe and well-tolerated, they have the potential to enhance the quality of life in a broad group of patients.

## 1 Introduction

In March 2020, COVID-19 was declared a worldwide pandemic by the World Health Organization (WHO). From the first recorded case of COVID-19 until today, over 450 million cases worldwide have been counted. Within a few months of the initial outbreak of this predominantly pneumatological disease, several patients reported long-lasting symptoms for weeks to months after their acute infection, later described as Long- or Post-COVID (1, 2). Currently, there is no clear consensus regarding the taxonomy to describe COVID-19 sequelae. However, the majority of the literature refers to the British National Institute for Health and Care Excellence (NICE) guidelines that categorize COVID-19 sequelae into three separate definitions: (i) “acute COVID-19” for symptoms during the first month after infection, (ii) “ongoing symptomatic COVID-19” describing symptoms from four to twelve weeks after infection, and lastly, (iii) “Post-COVID-19 syndrome” for unresolved signs and symptoms of COVID-19 twelve weeks after infection (3). The term Long-COVID refers to any signs and symptoms that persist or develop after an acute COVID-19 infection. This includes both “ongoing symptomatic COVID-19” (4 to 12 weeks) and “Post-COVID-19 syndrome” (12 weeks or longer) (3). In the past two years, additional guidelines for patients and physicians have been published to help cope with these yet not fully understood secondary disorders (4, 5). Note that in the German S1-Guidelines, there is a clear distinction between Long- and Post-COVID syndrome. Here, the authors refer to Long-COVID syndrome, when patients report persistent symptoms for more than four weeks after the acute infection and to Post-COVID syndrome, when symptoms persist for more than twelve weeks (4). However, in this review, we will refer to the NICE guidelines and use the term Long-COVID as a general term referring to both time periods, four to twelve weeks, as well as persisting symptoms for more than twelve weeks.

Due to the novelty and complexity of Long-COVID, progress in understanding and treating Long-COVID is still limited. Two of the most frequently observed symptoms of Long-COVID are fatigue and cognitive impairments (6). The fatigue syndrome describes an overwhelming feeling of exhaustion that is manifested both cognitively and physically and does not resolve with rest or sleep (7). It is also frequently reported in other pathological conditions such as multiple sclerosis (MS, 8), Parkinson´s disease (9), stroke (10), and chronic fatigue syndrome (CFS, 11). Among people with MS, fatigue has been identified as the most detrimental symptom to quality of life (12), as is now similarly described by people with Long-COVID (13, 14).

Despite its clinical importance, there is a lack of effective therapeutic methods to alleviate fatigue. However, in recent years, numerous replicated studies, primarily conducted in people with MS, have demonstrated the efficacy of transcranial direct current stimulation (tDCS) as a promising, non-invasive, and non-pharmacological method of reducing fatigue (see 15 for a systematic review). Additionally, studies using transcranial alternating current stimulation (tACS) or transcutaneous vagus nerve stimulation (tVNS) show promising results (16–18). Therefore, the aim of this review article is to give a more detailed insight into Long-COVID-related fatigue and to discuss non-invasive brain stimulation (NIBS) techniques as a possible alternative therapeutic approach. The review includes data before August 2022, covering the first two and a half years of COVID-19 research and approximately the first two years of Long-COVID research.

## 2 Long-COVID

The main risk factors for Long-COVID are the amount and duration of symptoms at the acute stage of infection, body mass index, and female sex (19–21). Thus, according to Sudre et al. (21), Long-COVID was more likely to occur when patients reported more than five symptoms within the first week of infection. These included fatigue, headache, dyspnea, hoarse voice, and myalgia. Interestingly, the likelihood and severity of Long-COVID symptoms are not associated with the initial severity of the acute COVID-19 infection (19, 22).

The severity of Long-COVID varies greatly among individuals, from mild disturbances that last a few weeks to severe illnesses that keep a person from managing their everyday lives for an extended period of time (6). Additionally, symptoms can be persistent or undulant, meaning that they are present at all times or disappear and repeatedly return (23). There is a considerable variation in the incidence rates of Long-COVID among studies. Thus, Sudre et al. (21) reported that 13.3% of the participants reported symptoms lasting for over 28 days, 4.5% for over eight weeks, and 2.3% for over twelve weeks. Another study by Nalbandian et al. (6), reported that up to 55% of COVID-19 patients might develop persisting symptoms after their acute infection. Furthermore, persistent symptoms after an acute COVID-19 infection do not necessarily lead to a reduction in quality of life. Thus, in a study by Giszas et al. (24), 70.7% of the participants reported persistent symptoms, while only one-third of those also reported that these symptoms significantly reduced their quality of life. Giszas et al. (24) therefore proposed to distinguish between Long-COVID “disease”, in which quality of life is significantly diminished and Long-COVID “condition”, in which quality of life is near-normal. In summary, while not entirely conclusive, these rates indicate that a significant amount of individuals who have had COVID-19 may develop Long-COVID.

The mechanisms behind the onset of Long-COVID are etiologically complex and can affect different organ systems within the body. Correspondingly, Long-COVID can entail a plethora of signs and symptoms months after the acute infection (23, 25). Neurological symptoms of Long-COVID primarily include cognitive and physical fatigue, as well as cognitive impairment, also referred to as brain fog (26). Other symptoms include headaches, gustatory and olfactory disturbances, neuropathic pain, and motor-sensory symptoms like sensitization disturbances such as numbness or paresthesia (26).

## 3 Long-COVID-related fatigue

People affected by Long-COVID report severe cognitive and physical fatigue as well as brain fog that make it almost impossible to execute daily tasks (23). Fatigue is therefore identified as the most detrimental symptom to the quality of life of people suffering from Long-COVID (14, 27).

The emergence of Long-COVID and its main symptom of severe fatigue has brought up a topic that researchers have tried to explore in its entity and have not yet fully understood. Thus, although fatigue is a common symptom in many neurological disorders, the exact etiology and underlying pathophysiology still remains unclear. It has been attributed to a variety of pathomechanisms, including primary causes such as axonal demyelination or atrophy (28–33), as well as neuroendocrine dysregulation (34, 35) or an underlying immune system malfunction (36, 37), see Palotai and Guttmann (38) or Chalah and Ayache (39) for comprehensive reviews.

A dysregulated immune system, as experienced in fatigue-typical neurological diseases as well as after an acute COVID-19 infection (40), may cause severe and ongoing damage to the brain, even in the absence of the primary viral attack. Thus, an acute COVID-19 infection leads to an overwhelming immune response called systemic inflammatory response syndrome (SIRS). During SIRS, the immune system is activated, and pro-inflammatory cytokines are excessively released, resulting in a variety of symptoms, among others, fatigue (41). To return to immunologic homeostasis, this process is then followed by a compensatory anti-inflammatory response (compensatory anti-inflammatory response syndrome, CARS), a mirror-imaged counter-regulation to SIRS that dampens the pro-inflammatory state and deactivates the immune system. However, if this balance between SIRS and CARS is dysregulated and the inflammatory response is repressed too far, this may lead to a stage of prolonged immunosuppression, provoking chronic pro-inflammatory cytokines production and an impaired immune competence, thereby making individuals highly susceptible to secondary infections (41–43). Inflammatory responses and, in consequence, anti-inflammatory reactions vary from person to person depending on the viral load they were exposed to, the adequacy or inadequacy of their immune system prior to an infection and whether or not they are dealing with comorbidities (41). Persistent inflammation and simultaneous suppression of the immune system have been consistently reported in people with Long-COVID (42) and also in CFS. Like Long-COVID, CFS is characterized by immune and nervous system disorders that lead to persistent difficulty in physical and mental functioning (44). It usually starts in previously healthy individuals who have overcome a viral infection (45), and it is characterized by persisting symptoms that include fatigue, post-exertional malaise, low blood pressure, cognitive disturbances, sleep problems, hypersensitivity and pain, as well as symptoms that can be classified under immune dysfunction (11).

In addition to the dysregulated immune system, reduced cerebral blood flow (CBF) has been described as another inflammation-related component in the development of fatigue in neurological diseases, CFS and Long-COVID. Hence, Fluge et al. (45) proposed a framework model for the initiation and maintenance of CFS, in which an initial inflammatory response leads to a release of B-cells and antibodies. These, in turn, can affect the vascular system and impede neurovascular control, resulting in impaired blood flow autoregulation and, ultimately, tissue hypoxia. Moreover, reactive oxygen species might play an important role in this process as they have consistently been implicated as an integral aspect of CFS pathophysiology (46, 47). Thus, initially released after an immune response, they trigger a chain reaction that induces a vasoconstrictor response, which is then followed by a decreased regional CBF (46–48). The decrease in CBF and hypoxia, as well as the body’s attempt to compensate for the impairment and maintain vascular homeostasis, lead to the symptoms of persistent fatigue that we see in the suffering individuals. Often, the effects of fatigue are particularly noticeable after physical or cognitive exertion, as the higher oxygen demand cannot be met (45). CBF decline has been reported in several studies regarding MS-related fatigue (49), CFS (50, 51) as well as LCOF (52, 53).

Additionally, as shown in MS-related fatigue, evidence exists for the involvement of a frontoparietal dysfunction and a malfunctioning cortico-striato-thalamo-cortical network in people with Long-COVID (54). Thus, an MRI- and PET-based study conducted by Hosp et al. (55) assessed the cognition of hospitalized COVID-19 patients shortly after their acute infection as well as six months after recovery. Directly after the acute stage, they found impaired memory and disturbed concentration. Interestingly, scans of COVID-19 patients showed less activity in neocortical areas with a predominant frontoparietal hypometabolism and, in reverse, higher activity in the brainstem and cerebellum. Additionally, this pattern correlated with the severity of impaired cognitive functions. At a 6-month follow-up, the frontoparietal hypometabolism was reversible but still significantly reduced compared to healthy controls (56). Similar metabolic changes are also found in people with MS who suffer from fatigue (57, 58).

## 4 General obstacles in fatigue research

A possible explanation for the lack of progress in understanding fatigue can be attributed to the lack of a universal definition and classification of fatigue. Therefore, we and others proposed a unified taxonomy that is disease-nonspecific and universally applicable (7, 59). Accordingly, the fatigue syndrome can be subdivided into physical, cognitive, and psychosocial fatigue (8). In this review, we focus on cognitive fatigue. Cognitive fatigue is defined as a decrease in cognitive resources and can further be described as a trait and a state component. Cognitive trait fatigue refers to an ongoing overall status of mental exhaustion that changes slowly over time and does not resolve with rest or sleep, while cognitive state fatigue refers to the subjectively perceived level of mental fatigue at a particular time (60). In recent years, it has become increasingly evident that, in addition to a subjective component, there is also an objectively measurable performance decline while executing a cognitively demanding task, which is often referred to as mental fatigue or fatigability (59).

However, the fatigue diagnosis is still very subjective. It is carried out using self-report questionnaires that assess the severity of trait fatigue and retrospectively ask subjects how they have been feeling over the past two to four weeks. Noteworthy, these questionnaires exclusively focus on the subjective experience of people suffering from fatigue and assess retrospective statements that are mood-sensitive and susceptible to psychological errors. Additionally, it has been reported that in people with MS, the questionnaires show low correlations among each other and assess different aspects of fatigue (61). Subjective state fatigue is typically assessed *via* numerical rating scales or visual analog scales that ask the patient to rate how exhausted they feel “right now at this moment”. To provide a comprehensive clinical diagnosis of fatigue, it is imperative to combine the assessment of subjective fatigue with objective evaluations of fatigue’s impact on physical or cognitive functioning. As behavioral parameters, primarily reaction time and accuracy changes are used. However, these show inconsistent results since participants are often able to maintain their performance despite a pronounced subjective feeling of exhaustion. In recent years, electrophysiological parameters, such as event-related potentials (62–64), sensory and sensorimotor gating deficits (65–67), and increasing fronto-medial theta and occipital alpha power (59, 68, 69) have been established as promising objective fatigability markers that are not subject to psychological biases. However, to this date, the relationship between subjective fatigue and fatigability is still a topic of controversy. Both may occur simultaneously or independently and are rarely associated (59). This discrepancy between subjectively perceivable fatigue and objectively measurable fatigability is also shown in recent Long-COVID studies. In a study by Dressing et al. (27), people with Long-COVID reported that their symptoms severely interfered with their daily lives, and 67% had subjective fatigue scores above the critical cutoff score for cognitive fatigue. However, an exhaustive assessment of their cognitive performance revealed only mild impairments and no objective changes in PET scans compared to healthy controls.

Due to the complex pathogenesis and the multifactorial character of fatigue, the search for an optimal therapy remains challenging. Possible treatment options can be divided into pharmacological and non-pharmacological treatments. Amantadine and Modafinil are considered possibly useful for the management of fatigue in people with MS. However, both drugs are currently prescribed off-label and show inconsistent results in fatigue improvement as well as attention and daytime sleepiness (70). Some studies show short-term fatigue improvements in non-pharmacological therapy options, such as physical training, energy conservation strategies, and cognitive-behavioral therapy (71, 72). Wearing a cooling vest (73) or staying in a cooled room for a short time (74) has been shown to bring about relief in some heat-sensitive subjects. Additionally, more general lifestyle changes are suggested, such as abstaining from smoking, reducing caffeine intake, and adjusting daily routines with scheduled breaks (70, 71).

However, clear success in fatigue therapy is still lacking. The treatment approaches previously discussed report controversial results and are based on small sample sizes. The heterogeneity of the symptoms and the complex pathogenesis make finding an optimal treatment strategy difficult. In recent years, NIBS has gained much attention as a promising non-invasive and non-pharmacological approach to fatigue treatment.

## 5 Non-invasive brain stimulation as a therapeutic option for fatigue

During NIBS, usually small electrical currents are applied. The shape, intensity, and duration of the applied current produces acute or long-lasting effects on the brain’s excitability, activity, and connectivity (75). NIBS modalities that have been widely used are transcranial direct current stimulation (tDCS), transcranial alternating current stimulation (tACS), and transcutaneous vagus nerve stimulation (tVNS). They offer the unique opportunity to manipulate the maladaptive neural activity underlying fatigue. The neuromodulatory potential of these modalities has already been widely shown in various neurological and psychiatric conditions, highlighting the potential for a clinical application of NIBS (76). When used and monitored according to the international safety guidelines (77), NIBS is considered safe and well-tolerated. Given its relatively low costs and risks, it can be made available to a broad range of people suffering from fatigue. Additionally, NIBS also has the considerable advantage that it can be used to specifically influence neuronal activity and directly observe resulting behavioral changes. Thus, direct causal relationships can be uncovered instead of showing only correlative relationships, which is of particular interest for a better understanding of pathomechanisms (78).

**Figure 1:**
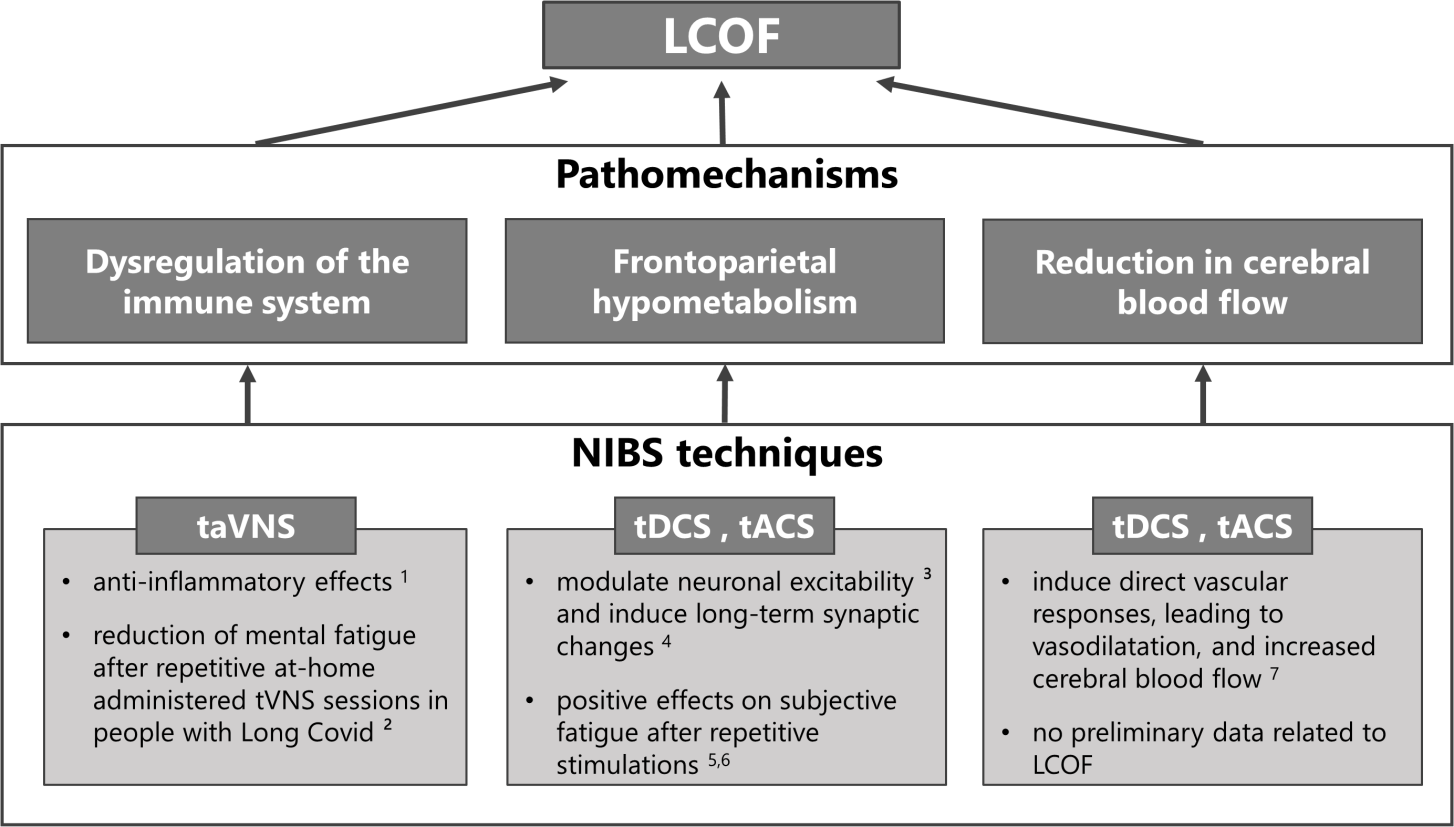
Non-invasive brain stimulation (NIBS) techniques for the treatment of individual pathomechanisms of Long-COVID-related fatigue (LCOF). Transcutaneous auricular vagus nerve stimulation (taVNS) has been shown to have anti-inflammatory effects through its efferent projections, the so-called cholinergic anti-inflammatory pathway (^1^ 81), and could have a stabilizing effect on the dysregulated immune-system after an acute COVID-19 infection that could lead to LCOF. First positive effects support this hypothesis and show a reduction of mental fatigue in people with LCOF after repetitive taVNS sessions (^2^ 16). Transcranial direct current stimulation (tDCS) and alternating current stimulation (tACS) have been shown to modulate neuronal responsiveness (^3^ 78) and to induce long-term effects *via* long-term potentiation (^4^ 82). They could therefore be used to counteract the observed frontoparietal hypometabolism after an acute COVID-19 infection that has been associated with LCOF. Preliminary data to support this hypotheses show positive effects on self-reported fatigue scores after repetitive sessions in people with LCOF (^5^ 83, ^6^ 84). Following neuronal acitivity or *via* direct vascular responses, tDCS and tACS have also been shown to increase cerebral blood flow (^7^ 85). Therefore they might also be an optimal strategy to counteract the observed blood flow reduction in people with LCOF. However, while several data exists that has shown increased cerebral blood flow after tDCS and tACS in healthy subjects and other neurological diseases, no data exists for LCOF.

NIBS techniques can be used in any of the previously described NICE categories to treat the different symptoms (see 79 and 80 for comprehensive reviews). The purpose of this review, however, is to explore the potential use of NIBS to treat LCOF, in particular cognitive fatigue. Therefore, we will provide a summary of studies that have addressed this topic in the following sections. An overview of the NIBS techniques proposed to treat LCOF is illustrated in **Figure 1**.

### 5.1 Transcranial direct current stimulation

During tDCS, two or more surface electrodes deliver a constant low-intensity electrical current through the scalp. The stimulation modulates the neuronal transmembrane potential and results in a shift of the resting membrane potential that leads to depolarization (anodal) or hyperpolarization (cathodal) of neuronal membranes (78, 86, 87). While the described modulation of cortical excitability is reversible (88), other studies have successfully demonstrated that the excitability-enhancing effects outlasted the stimulation period by several minutes to hours, possibly as a consequence of long-term synaptic changes occurring in the stimulated area (82, 86). Furthermore, these effects seem to be influenced by a variety of factors, such as electrode-to-cortex distance and the thickness of cerebrospinal fluid (89), as well as the orientation of pyramidal neurons (90).

As fatigue is a highly prevalent symptom of MS, most of the tDCS studies designed to treat fatigue have been conducted on people with MS (91–102). According to the pathogenesis of fatigue, mainly frontal, parietal (DLPFC, PPC), as well as sensorimotor regions (primary motor cortex M1, primary sensory cortex S1) were the target regions of the performed stimulations. Most of the studies applied 1.5 mA or 2 mA for a duration of 15 min, 20 min, or 30 min. The stimulations were applied for three, five, or ten consecutive days (see 59 or 15 for comprehensive reviews). Before and after the stimulations, subjective trait fatigue was assessed *via* self-reported questionnaires. Most of the studies reported significant improvements in subjective fatigue after anodal stimulation compared to sham stimulation. Interestingly, repetitive anodal tDCS stimulations independently administered at the patients´ homes using a remotely supervised stimulation protocol also led to significant improvements in fatigue ratings (97, 103). One study found no fatigue improvements in the overall study group, but the reduction in fatigue ratings was related to frontal lesion load in people with MS (100). TDCS effects on cognitive fatigability in people with MS were assessed in two studies (63, 99). Both studies examined time-on-task effects on reaction times and subjective state fatigue. Additionally, Fiene et al. (63) examined P300 EKP changes with time-on-task. Anodal tDCS over the right parietal cortex (99) or the DLPFC (63) counteracted the fatigability development in people with MS and led to decreased reaction times with time-on-task and greater P300 amplitudes as well as a reduced increase in P300 latencies.

Repetitive anodal tDCS stimulations over the left DLPFC also positively affected fatigue ratings in people with Parkinsons-related fatigue that lasted up to three months (104). Another study demonstrated that repetitive anodal tDCS sessions over the left DLPFC in a home-treatment context were well tolerated and positively affected subjective fatigue in people with Parkinsons-related fatigue (105), also see Zaehle (106) for a recent review.

In healthy participants, anodal tDCS over the left DLPFC reduced vigilance deficits caused by sleep deprivation and improved subjective state fatigue scores (107–109). Furthermore, anodal tDCS sustained or even improved the working memory performance in an hour-long two-back task (110). Recently, we showed that a single dose of anodal tDCS successfully counteracted fatigability development in healthy participants and, in turn, reduced the increase in occipital alpha power and the decline in sensory gating resulting from fatigability (66).

Several Case Reports already exist that present preliminary evidence for the effectiveness of tDCS on LCOF. Gómez et al. (83) administered 20 repetitive frontal tDCS stimulations in one Long-COVID patient and reported a clinically meaningful reduction in subjective trait fatigue ratings by 50 points. Furthermore, the patient felt less anxiety and showed improved cognitive performance. Another Case Report by Eilam-Stock et al. (84) reported two cases in which 15 repetitive, at-home administered frontal tDCS stimulations were applied in two Long-COVID patients. While Patient 1 reported significant improvements in fatigue perception, it remained stable in Patient 2. However, both patients returned to their job after treatment and resumed most of their prior activities.

Additionally, tDCS has been used to counteract pathological CBF declines. Thus, following neuronal activity or *via* direct vascular responses, tDCS can lead to vasodilatation, thereby increasing CBF (85). TDCS-effects on CBF have been demonstrated to be dose- and polarity-dependent (111, 112). Hence, anodal tDCS increased CBF with higher increases at greater intensities (111, 113, 114), whereas cathodal tDCS decreased CBF (111, 112). However, a high interindividual variability exists (112). Additionally, it has again been demonstrated that repetitive sessions have cumulative effects (113, 114).

### 5.2 Transcranial alternating current stimulation

The tACS method involves the delivery of rapidly alternating electrical currents through the scalp. It is designed to induce periodic shifts in membrane potentials and entrainment of neural activity to the frequency of stimulation (115, 116).

In healthy subjects, fatigability has been related to a systematic shift from fast to low-frequency waves that repeatedly resulted in increased frontomedial theta power as well as increased occipital alpha power (62, 66, 117–119). Based on correlational studies, Clayton et al. (68) proposed an oscillatory model of sustained attention. The authors suggest that in a fatiguing brain, the increase in frontomedial theta power is a consequence of compensatory mechanisms to enhance top-down control processes, whereas the increase in alpha power in task-relevant cortical areas suppresses information processing, ultimately leading to attention deficits.

The ability to modulate abnormal oscillations using tACS motivates the possible clinical application of tACS stimulation for an effective fatigue treatment. According to the model by Clayton et al. (68), frontomedial theta power plays a central role in monitoring cognitive processes while performing a cognitively demanding task. Hence, the frontomedial application of theta-tACS might increase frontal cognitive control and counteract performance decline with time-on-task. Moreover, the tACS-induced frontomedial theta increase may enhance attention by suppressing fatigability-related occipital alpha power *via* fronto-posterior phase synchronization (68).

However, the use of tACS to treat fatigue has been sparse and restricted to healthy subjects. One study by Loffler et al. (17) applied 40-Hz gamma-tACS over the visual cortex during a 60-minute vigilance task. Their goal was to counteract the fatigability-related increase of inhibitory alpha power over task-relevant cortical regions. Gamma-tACS was able to counteract the reaction time increase with time-on-task. However, effects on subjective fatigue remain speculative due to the lack of subjective ratings.

One study investigated the effects of 40-Hz gamma-tACS on CBF in people with Alzheimer´s disease (18). The authors administered one-hour-long daily stimulations over the course of two to four weeks. Results showed a significant increase of CBS in the stimulation area (temporal lobes) after the tACS treatment. Additionally, cognitive performance was improved.

### 5.3 Transcutaneous vagus nerve stimulation

Another NIBS technique of interest is tVNS, particularly transcutaneous auricular VNS (taVNS). In taVNS, the auricular branch of the vagus nerve is stimulated that bilaterally innervates the human ear, resulting in a specific modulation of various brain structures connected with the vagus nerve (16, 120). The vagus nerve is able to modulate inflammatory responses through its efferent projections, the so-called cholinergic anti-inflammatory pathway (CAP; 81, 121), and activation of the CAP attenuates neuroinflammation (122, 123). Accordingly, electrical stimulation of the vagus nerve has been shown to be effective in reducing inflammatory responses in rheumatoid arthritis (124, 125) and inflammatory bowel disease (IBD; 126). Analogously, non-invasive taVNS can reduce acute inflammatory responses (127, 128) by activating the CAP (129), while the method is generally considered safe and well-tolerated (130). Consequently, taVNS has been demonstrated to have anti-inflammatory, anti-pain and anti-depressant effects in patients with Long-COVID (16). Thus, Badran et al. (16) investigated the effects of repetitive, at-home administered taVNS sessions on the fatigue syndrome in people with Long-COVID and showed a positive mild to moderate effect on mental fatigue in a subset of individuals.

In summary, the previous literature has shown that a single dose of tDCS or tACS is an effective therapeutic option for treating subjective fatigue and fatigability with time on task. Furthermore, it has been demonstrated that repetitive sessions have cumulative effects on fatigue and that the effects outlast the stimulation period. The previously described frontoparietal hypometabolism, as well as the reduction in CBF in Long-COVID patients, indicate similar pathomechanisms of fatigue in Long-COVID and fatigue in other neurological diseases. Furthermore, tVNS might be used to restore the dysregulated immune system in Long-COVID. However, it is important to note that the reported positive effects of all NIBS methods in various healthy, as well as clinical subgroups, may not translate to LCOF. Thus, the pathogenesis of fatigue, in general, is poorly understood, and it appears that LCOF greatly overlaps with CFS-related fatigue. In addition, LCOF is also more volatile and less predictable than in people with MS. Nevertheless, reliable and valid positive effects of NIBS applications are emerging, and there are first promising results related to LCOF. Consequently, there is a reasonable possibility that all NIBS methods presented here in this review may improve subjective fatigue perception as well as fatigability-related performance declines in people with Long-COVID, as has already been described in preliminary data (see **Figure 1**).

## 6 Conclusions

There are a large number of people who describe persistent symptoms after acute COVID-19 infection and are eventually diagnosed with Long-COVID. Fatigue is the most frequently observed symptom and represents a major threat to the medical health care system. In this review, we presented three NIBS methods that have the ability to modulate maladaptive fatigue-related neuronal activity that is shown in fatigued patients of several neurological diseases but also recently in people suffering from Long-COVID. Many of the presented studies have already been shown to improve fatigue, in particular cognitive fatigue, in healthy subjects as well as subjects with neurological disorders such as multiple sclerosis or Parkinson’s disease. Additionally, preliminary data also suggest positive effects on LCOF. Future studies need to systematically determine the parameters of an optimal stimulation setting, specifically pay attention to an established fatigue taxonomy and complement the effects on subjective fatigue with an objective and valid assessment of fatigability in Long-COVID. In the absence of an effective fatigue therapy, neuromodulation by NIBS provides a promising alternative treatment approach. The methods are safe and well-tolerated and allow for large-scale use in clinical practice.

## Author contributions

SL and TZ conceived the idea. SL designed, coordinated, and wrote the review: LK wrote individual sections of the review. AH and TZ revised the manuscript and contributed to further drafts. All authors contributed to the final version of the manuscript. All authors contributed to the article and approved the submitted version.
